# Data of drowning related deaths with reference to entomological evidence from Haryana

**DOI:** 10.1016/j.dib.2017.10.064

**Published:** 2017-11-02

**Authors:** Jyoti Dalal, Sapna Sharma, Kapil Verma, S.K. Dhattarwal, Tapeshwar Bhardwaj

**Affiliations:** a Department of Genetics, Maharshi Dayanand University, Rohtak, Haryana, India; b Department of Forensic Medicine, Post Graduate Institute of Medical Sciences, Rohtak, Haryana India

**Keywords:** Drowning, Entomological evidences, PMI

## Abstract

Most often the newspapers and bulletin come out with voluminous cases of deaths due to drowning. At the same time an ample section of such cases encompass entomological evidences, that can be scrutinized as a very useful parameter in estimating post mortem interval (PMI). This research database is an outcome of a 2 years reflective study, based on an assessment of records related to human deaths due to drowning. The drowned bodies from various districts of Haryana are sent to PGIMS (Post Graduate Institute of Medical Sciences), Rohtak. The study took the data of year 2015–2016 into consideration. All the cases were reviewed and summarized in terms of monthly occurrence of total cases, age differentiation, gender differentiation and month wise occurrence of entomological evidences on the dead bodies through detailed study of post mortem findings. This data will lead to an insight into the magnitude of drowning deaths in Haryana along with the usage of entomological data for determining Post Mortem Submersion Intervals (PMSI).

**Specifications Table**

**Table:** 

Subject area	Forensic Science
More specific subject area	Drowning deaths and Forensic Entomology
Type of data	Figure and Tables
How data was acquired	Data has been acquired by survey method from Post Graduate Institute of Medical Sciences (PGIMS), Rohtak (Haryana) with kind permission of the concerned authority.
Data format	Filtered and Analyzed
Experimental factors	Nil
Experimental features	The distribution patterns of death due to drowning in aquatic system were evaluated in the context of location, year, month, gender and age wise variations with respect to the presence or absence of entomological evidence.
Data source location	Rohtak, India
	Latitude: 28.895515 and Longitude: 76.606613
	GPS coordinates: 28° 53′ 43.8540′′ N and 76° 36′ 23.8068′′ E.
Data accessibility	Data is available with this article

**Value of the data**

• Routine monitoring of deaths due to drowning in an aquatic environment is very important for medicolegal and forensic purposes around the world.
• Little is known about the trend of drowning related death in an aquatic habitat with respect to entomological evidence, so the present data emerge of immense value since no data is available on this important aspect from Haryana.
• The information provided in this data set bring about new ideas for forensic experts and police officers about the trend of drowning deaths in an aquatic habitat with respect to the presence or absence of any entomological testament.
• This kind of data is also useful for scientists and researchers working in the field of forensic medicine, aquatic entomology and forensic entomology. It administers detailing to the inhabitance of entomological specimen in this particular area.
• Entomological facts generated in the study provide a pandora of information which can be useful for future efforts in the discipline of forensic entomology.
• The details contributed here would be the first of its kind for the readers to fully understand the types and trends of drowning deaths with a special emphasis on entomological knowledge in an aquatic environment.
• The present data shows the presence of entomological specimen with most of the recovered drowned bodies. The neglect of this evidence is no more possible.
• This data will also provide means of determining PMI in aquatic environments, when the initial signs of decomposition have passed off.
• This data serves as a source of information for the research institutes as well as for the forensic science community. It can be further used to correlate the significance of entomological specimen present in drowned bodies for future research in this particular area.

## 1 Data

**Fig. 1:**
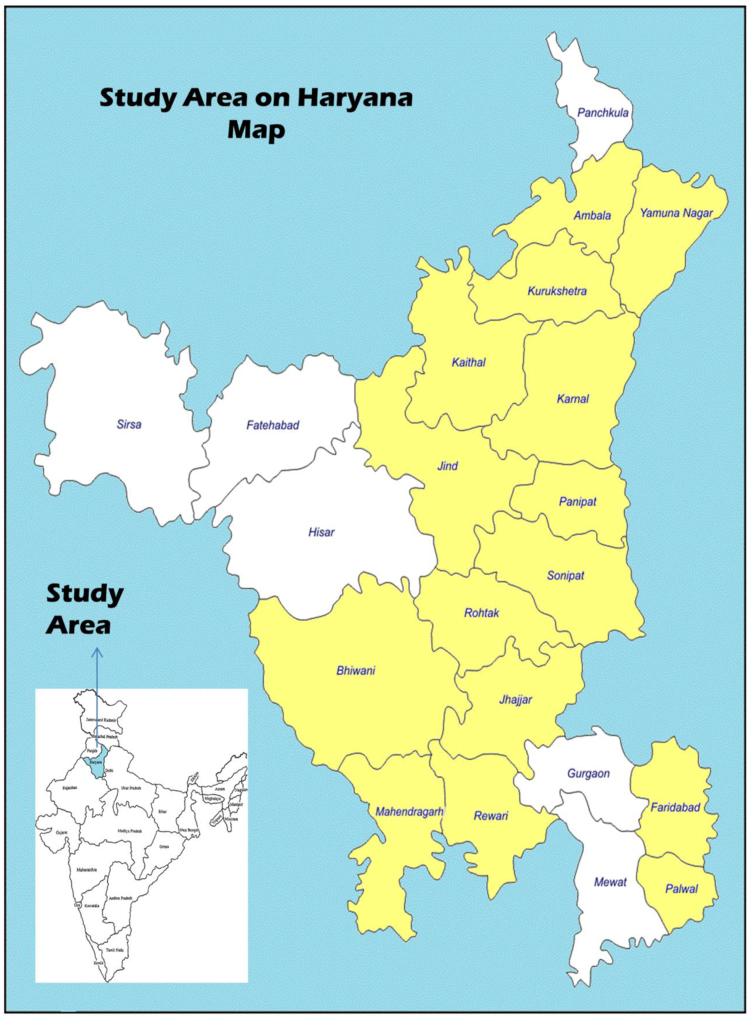
Map of Haryana showing study area.

The data provided in this article explain about the trend of deaths in an aquatic environment and the significance of entomological evidence in drowning deaths from Haryana, India (Fig. 1). There have been nearly no studies to correlate drowning deaths with entomological specimens. Forensic entomology is an upcoming and promising science in India but also a neglected one [1]. This study proves the association of insects species on drowned bodies. The initial decomposition signs usually pass off, so this branch becomes of special significance for ascertaining a minimum Post Mortem Submersion Interval (PMSI) if used efficiently.

## 2 Experimental design, materials and methods

Experimental design consists of frequencies based on the distribution patterns of death due to drowning in an aquatic system. These patterns were evaluated in the context of location, year, month, gender and age wise variations. The present database comprises of drowning death cases reported in the Department of Forensic Medicine, Post Graduate Institute of Medical Sciences (PGIMS), Rohtak from 1st January, 2015 up to 31st December, 2016. All the drowning related death cases were surveyed and filtered from the Post-mortem Reports (PMRs) (Table 1, Table 2, Table 3, Table 4, Table 5).

**Table 1:** Drowning cases reported from different districts in 2015–16.

**Name of district**	**2015**	**2016**
Ambala	0	2
Bhiwani	21	32
Faridabad	12	14
Jhajjar	32	41
Jind	6	22
Kaithal	6	10
Karnal	22	24
Kurukshetra	0	2
Mahendergarh	7	5
Palwal	0	1
Panipat	21	28
Rewari	9	18
Rohtak	30	42
Sonepat	0	1
Yamunanagar	1	2
Total	167	244

**Table 2:** Month wise observation of drowning cases in 2015–16.

**Month**	**2015**		**2016**	
	**Number of cases**	**Percentage (%)**	**Number of cases**	**Percentage (%)**
January	4	2.39	9	3.69
February	6	3.59	10	4.1
March	5	2.99	16	6.56
April	8	4.79	25	10.24
May	25	14.97	28	11.48
June	20	11.97	29	11.89
July	23	13.78	29	11.89
August	29	17.36	38	15.58
September	21	12.57	23	9.42
October	11	6.58	21	8.6
November	9	5.38	7	2.86
December	6	3.59	9	3.69
Total	167		244

**Table 3:** Distribution frequency of drowning deaths in 2015–16.

	**2015**		**2016**	
	**Number of cases**	**Percentage (%)**	**Number of cases**	**Percentage (%)**
Male	135	80.84	198	81.15
Female	30	17.96	44	18.03
Unidentified	2	1.2	2	0.82
Total	167		244

**Table 4:** Gender wise frequency of drowning deaths according to different age groups in 2015–16.

**Age group**	**Males**				**Female**			
	**2015**		**2016**		**2015**		**2016**	
	**Number of cases**	**Percentage (%)**	**Number of cases**	**Percentage (%)**	**Number of cases**	**Percentage (%)**	**Number of cases**	**Percentage (%)**
0–15	5	3.7	6	3.03	1	3.33	3	6.82
16–30	36	26.67	63	31.82	16	53.33	25	56.82
31–40	44	32.6	51	25.76	10	33.33	8	18.18
41–60	32	23.7	51	25.76	1	3.33	5	11.36
>60	7	5.18	21	10.6	2	6.67	3	6.82
Age not mentioned	11	8.15	6	3.03	0	0	0	0
Total	135		198		30		44

**Table 5:** Data associated with drowning deaths with respect to presence or absence of entomological evidences in 2015–16.

**Month**	**2015**		**2016**	
	**With entomological evidences**	**Without entomological evidences**	**With entomological evidences**	**Without entomological evidences**
January	0	4	3	6
February	2	4	3	7
March	4	1	15	1
April	6	2	18	7
May	22	3	23	5
June	18	2	29	0
July	21	2	28	1
August	27	2	34	4
September	16	5	15	8
October	11	0	17	4
November	6	3	4	3
December	4	2	3	6
Total:	137	30	192	52
