# Supplementary material for: Data of drowning related deaths with reference to entomological evidence from Haryana
Source: Data Brief. 2017 Nov 2;15:975–80. doi: 10.1016/j.dib.2017.10.064 (PMC5684095; doi:10.1016/j.dib.2017.10.064)
Supplement: Supplementary file 1 — Transparency document [file mmc1.docx]

**Conflict of interests:** None declared
